# Inflammatory bowel disease and celiac disease: A bidirectional Mendelian randomization study

**DOI:** 10.3389/fgene.2022.928944

**Published:** 2022-08-19

**Authors:** Yue Shi, Sijia Feng, Mengdie Yan, Shuyan Wei, Kejia Yang, Yue Feng

**Affiliations:** Chengdu University of Traditional Chinese Medicine, Chengdu, China

**Keywords:** inflammatory bowel disease, Crohn’s disease, ulcerative colitis, celiac disease, Mendelian randomization

## Abstract

**Objective:** Although previous epidemiological studies have reported substantial links between inflammatory bowel disease (IBD), including Crohn’s disease (CD) and ulcerative colitis (UC), and celiac disease (CeD), the causal relationship between the two remains unknown. The purpose of the current study was to evaluate the bidirectional causation between IBD and CeD using Mendelian randomization (MR).

**Method:** We obtained genome-wide association study (GWAS) summary data of IBD (CD and UC) and CeD of thoroughly European ancestry from the IEU GWAS database. We screened eligible instrumental variables (IVs) according to the three assumptions of MR. MR was performed using MR-Egger, weighted median (WM), and inverse variance weighted (IVW) methods. The MR-Egger intercept and MR-PRESSO method investigated the horizontal pleiotropy effect. A leave-one-out analysis was performed to prevent bias caused by a single SNP.

**Results:** The study assessed a bidirectional causal effect between CD and CeD; CD increased the risk of CeD (IVW odds ratio (OR) = 1.27, 95% confidence interval (CI) = 1.19–1.35, *p* = 3.75E-13) and vice-a-versa (IVW OR = 1.09, 95% CI = 1.05–1.13, *p* = 1.39E-05). Additionally, CeD was influenced by IBD (IVW OR = 1.24, 95% CI = 1.16–1.34, *p* = 9.42E-10) and UC (IVW OR = 0.90, 95% CI = 0.83–0.98, *p* = 0.017). However, we observed no evidence of a causal relationship between CeD and IBD (IVW OR = 1.00, 95% CI = 0.97–1.04, *p* = 0.900) or UC (IVW OR = 0.96, 95% CI = 0.92–1.02, *p* = 0.172).

**Conclusion:** The present study revealed that IBD and CeD have a bidirectional causal relationship. However, it is slightly different from the results of previous observational studies, recommending that future studies focus on the mechanisms of interaction between CD and CeD.

## Introduction

The incidence of inflammatory bowel disease (IBD), characterized by persistent diarrhea, stomach discomfort, and perianal hemorrhage, is increasing worldwide. Ulcerative colitis (UC) and Crohn’s disease (CD) are the two main types of IBD (Hodson, 2016). In addition to intestinal symptoms, such as diarrhea and stomach discomfort, patients with IBD frequently experience extraintestinal symptoms (Rogler et al., 2021). Over the next few decades, the number of people with IBD is predicted to increase exponentially worldwide, providing enormous challenges to healthcare systems globally (Loftus, 2004; Ng et al., 2017; Mak et al., 2020). CeD is an autoimmune condition that affects people genetically prone to gluten immunological response (Lebwohl et al., 2018). CeD is more common in developed countries (Lebwohl et al., 2018); it was once assumed to exist only in Northern Europe and Australasia (Kang et al., 2013; Dhawan et al., 2019). However, it is now a worldwide illness, affecting 0.4% of the population in South America, 0.5% of the population in Africa and North America, 0.6% of the population in Asia, and 0.8% of the population in Europe and Oceania (Singh et al., 2018). CeD is becoming more prevalent due to improved diagnosis and testing, but the actual cause is an increase in the prevalence of immune-based diseases (Lebwohl and Rubio-Tapia, 2021).

IBD and CeD are autoimmune diseases involving damage to the gastrointestinal system. Both diseases are mainly caused by genes, environment, and gut microbes (Pascual et al., 2014). IBD only has 12% genetic overlap with CeD and it is not sufficient to explain their relationship. IBD and CeD both require an external trigger to start the disease process by activating the immune system. The genes shared by IBD and CeD are mainly for immune cell differentiation and signaling. Different immune-mediated pathways do exist, though, such as Th17 response that appears to be only relevant to IBD and Th1 for CeD. They have become more prevalent in recent decades, and they are gradually spreading from developed countries to the world. IBD and CeD may be caused by the same etiological factor. Clarifying the causal link between IBD and CeD is critical since there are more clinical reports of co-morbidity between the two diseases and their etiologies and clinical presentations are comparable. Observational research, on the other hand, came to diverse conclusions. Some studies consider a link between the two disorders (Tursi et al., 2005; Yang et al., 2005), whereas others disagree (Rönnblom et al., 2015). Several meta-analyses published recently reported similar findings (Shah et al., 2019; Pinto-Sanchez et al., 2020). Although prior studies have demonstrated a relationship between IBD and CeD, no evidence is reported that the two diseases are linked in a causative association.

Some limitations of traditional epidemiological research are confounding variables and reverse causality. These prior observation data were limited for link inference because of the probable bias produced by confounding factors; even this conclusion is debatable. For example, the gut microbiota is a complex and dynamic collection of environmental microbial communities that inhabit the human gastrointestinal tract (García-Santisteban et al., 2020; Zhang et al., 2021). Nevertheless, if exposure does not have a causal link with an outcome, public health or medical interventions focused on the exposure will be useless and waste resources. Even while randomized controlled trials (RCT) may be able to determine the existence of a causal association between IBD and CeD, they are time-consuming and expensive. Mendelian randomization (MR) tests this hypothesis when assessing if a link between a risk factor and an outcome can be explained by a causal impact (Davey Smith and Hemani, 2014). The Mendelian law of ‘random assignment of parental alleles to offspring’ is used in MR to simulate the random assignment of RCTs. Through genetic polymorphisms and allelic randomization, it is possible to circumvent the confounding challenges and reverse causality inherent in observational research, providing better evidence than traditional observational research when inferring cause-effect relationships (Davies et al., 2018).

**FIGURE 1:**
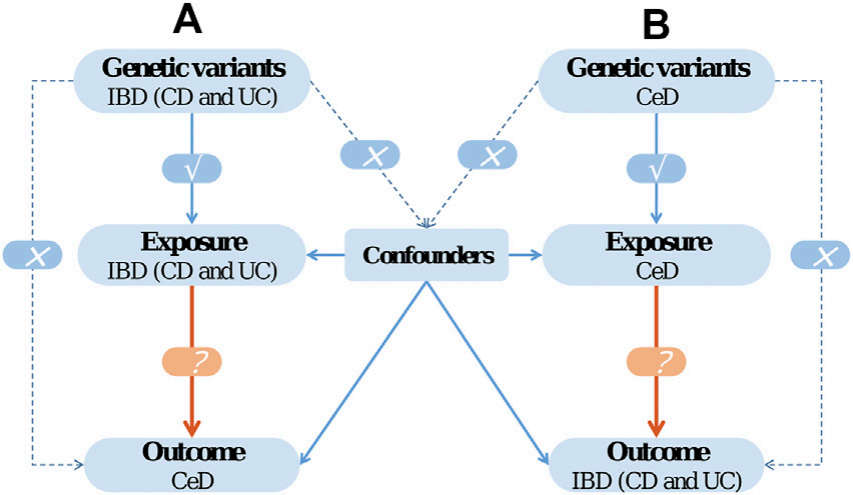
The design of bidirectional Mendelian randomization (MR) study. The '×' means that genetic variants are not associated with confounders or cannot be directly involved in outcome but *via* the exposure pathway. The '√' means that genetic variants are highly correlated with exposure.

In the current work, we conducted a two-sample bidirectional MR analysis to infer causality between IBD (CD and UC) and CeD. The bidirectional MR design is illustrated in Figure 1.

## Methods

### Study design overview

We used a two-sample MR method to quantify causal effects in both directions. In brief, we began by estimating the causative effects of IBD on CeD and then we moved on to evaluating the causative impact of CeD on IBD. The study contains three key stages to verify each inference direction: Selecting appropriate genetic IVs for the corresponding exposure, using different MR approaches, and evaluating pleiotropic effect and heterogeneity and sensitivity analyses.

### Data sources

To avoid the effects of population stratification, all SNPs and their associated summary data were acquired from studies that solely included populations of European ancestry. The IEU GWAS database (https://gwas.mrcieu.ac.uk/datasets/) contains GWAS summary statistics for IBD (including CD and UC), which may be downloaded. The study of IBD involved 96,486 people, with 89.8% of them having European heritage and the remaining 10% having East Asian, Indian, or Iranian backgrounds (Liu et al., 2015). We only included individuals of European origin for data analysis. The study comprised 12,882 participants with IBD and 21,770 people who were considered healthy controls. A total of 5,956 patients and 14,927 controls were included in the CD analysis; 6,968 individuals with UC and 20,464 healthy controls were included in the study.

We also searched for all CeD-related genome-wide association studies (GWASs) published in the publicly available IEU GWAS database. This study comprised 12,041 patients with CeD and 12,228 control subjects (Trynka et al., 2011). We eliminated the dataset with the Indian origin to ensure that the ethnicity was of European origin. Therefore, 11,812 patients with CeD were identified, and 11,837 control subjects were included.

All the data were gathered from previously published research available to the public. Thus, the study did not require ethical approval or patients’ consent.

### Instrumental variable selection

**FIGURE 2:**
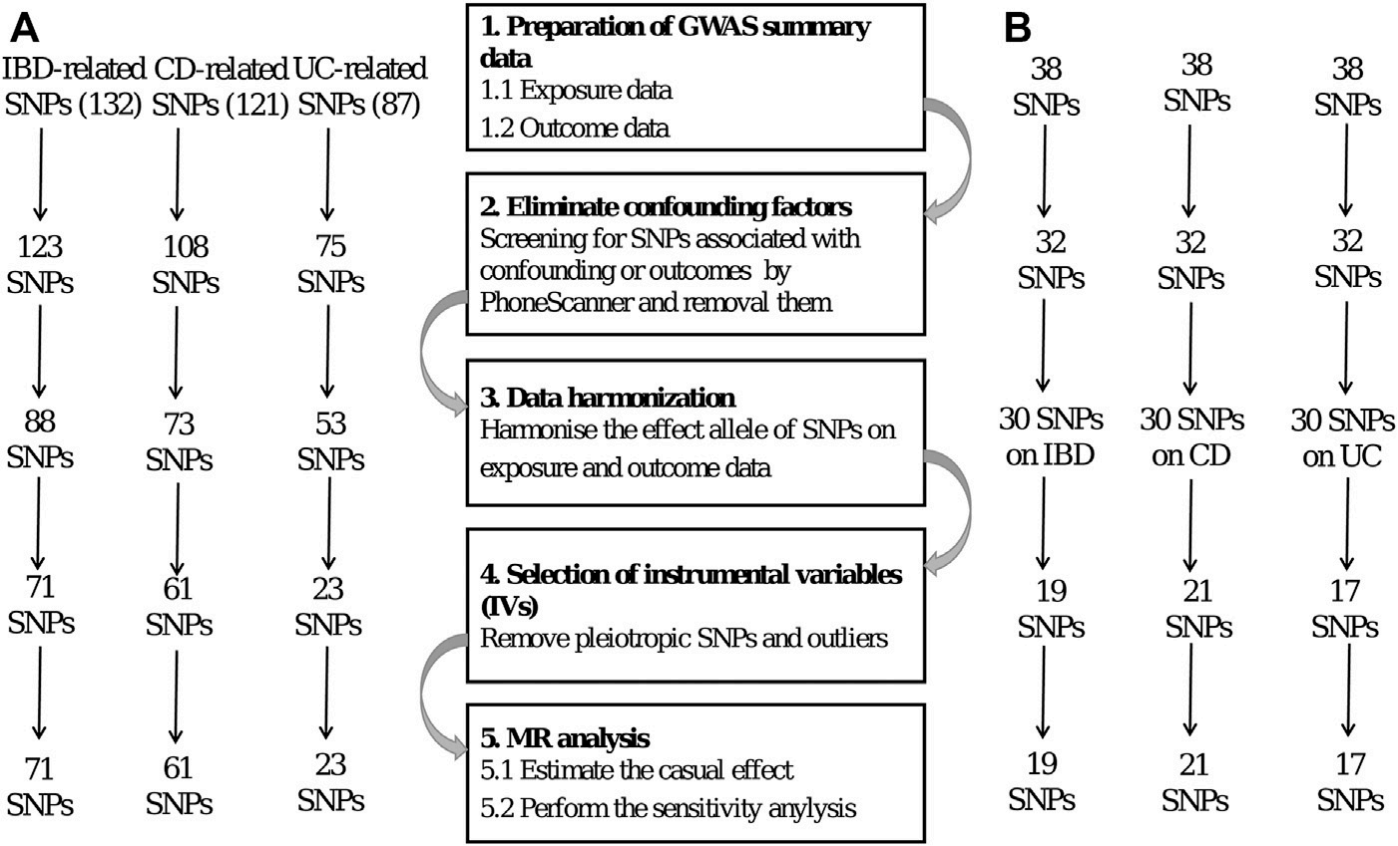
**(A)** and **(B)** represent exposure to IBD and CeD, respectively.

Genetic variations are used as instrumental variables (IVs) in MR. The researchers employ two separate samples to investigate the impact of IVs on exposures and outcomes, using publicly accessible information from extensive GWAS to guide their work. Several prerequisites must be met to obtain unbiased results (Hartwig et al., 2016), including the following: 1) the IVs should have a statistically significant link to the exposure; 2) the IVs should not be associated with confounders related to the exposure or the outcome, particularly outcome; and 3) the IVs should only have an effect on the outcome through exposure. The SNPs were selected as IVs based on the three assumptions of MR. Figure 2 shows a flow diagram depicting the selection of SNPs used to evaluate genetic instruments and conduct MR analysis.

First, we identified single-nucleotide polymorphisms (SNPs) associated with the exposure that were observed to be statistically significant across the genome (*p* < 5 × 10^–8^) to meet the first assumption. Furthermore, these SNPs exhibited a certain likelihood of mutation (minor allele frequency; MAF >5%). We used a clumping technique with R^2^ < 0.001 and a window size of 10,000 kb to eliminate SNPs associated with significant linkage disequilibrium (LD).

Second, we examined the Phenoscanner database (http://www.phenoscanner.medschl.cam.ac.uk/) for all SNPs linked with exposure to see whether there were any SNPs associated with confounding variables and outcomes (*p* < 5 × 10^–8^). These SNPs were manually eliminated to meet the assumptions of genetic instrumental variables being independent of outcome and confounding factors. In addition, we used the MR-Pleiotropy Residual Sum and Outlier (MR-PRESSO) approach to remove outlier variations to account for horizontal pleiotropy in our results.

Furthermore, we used the following equation to determine the F statistics (CRP CHD Genetics Collaboration, 2011) for SNPs cumulatively: F = (N−k−1)R^2^/k(1−R^2^). The variation of exposure explained by each IV is denoted by R^2^. F-statistics were used to assess the instrument’s strength (a device with an F value <10 was considered weak).

### Mendelian randomization analysis

All the analyses were performed in RStudio version 4.0.3 with the packages ‘TwoSampleMR’ and ‘MRPRESSO.’

Various MR approaches were used in our investigation to confirm the causal link between IBD and CeD, including the inverse variance weighted (IVW), the weighted median (WM), and the Mendelian randomization-Egger (MR-Egger) methods, with the IVW method dominating. The IVW method consistently estimates the causal influence of exposure on the result and offers the highest statistical validity (Burgess et al., 2013). However, when all the genetic variations are incorrect instrumental factors, Egger’s test still provides a valid null causal hypothesis test and a consistent causal impact estimate (Bowden et al., 2015). The WM technique can yield a consistent estimate when some genetic instruments are not authentic instrument variables (Bowden, et al., 2016a). The random-effects IVW method was used to integrate the causal effects of individual SNPs. Two additional methods were used to verify the credibility of the results. The odds ratio (OR) and 95% confidence interval (CI) evaluated the relative risk induced by the illness of interest.

For quality control, pleiotropy, heterogeneity, and sensitivity analyses were used. We used the MR-Egger regression to assess whether horizontal pleiotropy existed. Additionally, the MR-PRESSO test was conducted to determine whether pleiotropy was present (Verbanck et al., 2018) and manually remove SNPs which were outliers. The IVW method and egger regression were used to assess heterogeneity, and Cochran’s Q statistic was used to quantify it (Bowden, et al., 2016b). The leave-one-out approach recalculates the results by deleting individual SNP one at a time to verify the robustness of the results. We removed specific SNPs which have a disruptive effect on the results.

## Results

### Causal effects of inflammatory bowel disease on celiac disease

After stringent exclusion criteria, we included 71 SNPs for IBD, 61 for CD, and 23 for UC. All three IVs had F-statistics > 10, indicating that no weak instrumental variable bias existed.

Table 1 shows the MR estimations from various approaches. The Cochran’s Q test revealed heterogeneity (*p* < 0.05). As a result, we used the IVW approach in a random-effects model. We observed a significant causal relation between IBD and CeD (IVW OR = 1.24, 95% CI = 1.16–1.34, *p* = 9.42E-10). The results of WM were consistent with that of IVW (WM OR = 1.19, 95% CI = 1.10–1.29, *p* = 1.96E-05). The MR-Egger genetically predicted that IBD had no causal influence on CeD (MR-Egger OR = 1.03, 95% CI = 0.82–1.28, *p* = 0.813). The IVW estimates were consistent with the WM estimates. Because the test of horizontal pleiotropy was not statistically significant (*P* for MR-Egger intercept >0.05), the IVW estimates may be more robust than the MR-Egger regression estimates in terms of estimating causal effects. As a result, we recognized the IVW results and considered the causal impact of IBD on CeD risk.

**TABLE 1:** MR analysis of the causal association between IBD and CeD.

Exposures	Outcomes	nSNPs	Method	OR (95%CI)	P	Heterogeneity test			Pleiotropy test	F
						Method	Q	P	P intercept	
IBD	CeD	71	MR-Egger	1.03 (0.82–1.28)	0.813	MR-Egger	130.6	<0.001	0.080	29.6
			WM	1.19 (1.10–1.29)	1.96E-05	IVW	136.6	<0.001		
			IVW	1.24 (1.16–1.34)	9.42E-10					
CD	CeD	61	MR-Egger	1.21 (0.99–1.49)	0.066	MR-Egger	128.8	<0.001	0.681	31.5
			WM	1.26 (1.17–1.35)	1.13E-09	IVW	129.2	<0.001		
			IVW	1.27 (1.19–1.35)	3.75E-13					
UC	CeD	23	MR-Egger	1.06 (0.79–1.40)	0.716	MR-Egger	8.8	0.991	0.284	21.5
			WM	0.94 (0.84–1.06)	0.311	IVW	10	0.986		
			IVW	0.90 (0.83–0.98)	0.018					
CeD	IBD	19	MR-Egger	0.99 (0.94–1.05)	0.800	MR-Egger	48.3	<0.001	0.687	54.1
			WM	0.99 (0.97–1.02)	0.683	IVW	48.7	<0.001		
			IVW	1.00 (0.97–1.04)	0.900					
CeD	CD	21	MR-Egger	1.08 (1.01–1.15)	0.030	MR-Egger	46.1	<0.001	0.854	49.3
			WM	1.08 (1.03–1.13)	5.83E-04	IVW	46.2	<0.001		
			IVW	1.09 (1.05–1.13)	1.39E-05					
CeD	UC	17	MR-Egger	1.01 (0.91–1.11)	0.877	MR-Egger	37.3	0.001	0.321	35.5
			WM	0.96 (0.92–1.00)	0.078	IVW	40	<0.001		
			IVW	0.96 (0.92–1.02)	0.172					

IBD, inflammatory bowel disease; CD, Crohn’s disease; UC, ulcerative colitis; CeD, celiac disease; IVW, inverse variance weighted; WM, weighted median; nSNPs, number of SNPs used in MR; OR, odds ratio; CI, confidence interval.

Moreover, we discovered a substantial link between CD and CeD (IVW OR = 1.27, 95% CI = 1.19–1.35, *p* = 3.75E-13; WM OR = 1.26, 95% CI = 1.17–1.35, *p* = 1.13E-09). The OR was not different from the IVW and WM, despite the MR-Egger suggesting no statistical significance (MR-Egger OR = 1.21, 95% CI = 0.99–1.49, *p* = 0.066). The results exhibited an elevated level of confidence. The IVW method revealed statistical significance in the causal effect of UC on CeD (IVW OR = 0.90, 95% CI = 0.83–0.98, *p* = 0.017), with an OR < 1, indicating that UC was a protective factor for CeD. However, neither the WM nor MR-Egger techniques were statistically significant, and all ORs did not trend in the same direction, indicating that the results should be regarded with caution.

### Causal effects of celiac disease on inflammatory bowel disease

In reverse causality, CeD is the exposure factor used to demonstrate the outcome of IBD. We identified 19, 21, and 17 SNPs as IVs for the three distinct outcomes of IBD, CD, and UC, respectively. The F-statistics for all IVs were >10, suggesting that no bias of weak instrumental variable existed.

Similarly, we used a random effects model because of the considerable heterogeneity. According to the IVW method, CeD has no causal influence on IBD (IVW OR = 1.00, 95% CI = 0.97–1.04, *p* = 0.900). In addition, we could not discover any evidence of a link between CeD and UC (IVW OR = 0.96, 95% CI = 0.92–1.02, *p* = 0.172). CeD has a causal effect on CD (IVW OR = 1.09, 95% CI = 1.05–1.13, *p* = 1.39E-05). Other MR estimates provided equivalent results (Table 1).

All MR Egger regression yielded negative results (*P* for MR-Egger intercept >0.05), indicating no bias for horizontal pleiotropy. Supplementary Figures provide scatter plots, funnel plots, and leave-one-out sensitivity analysis. Although we excluded SNPs with a subversive impact, the leave-one-out sensitivity analysis revealed that a few SNPs had a influence on the results that have been combined, which might be a cause of heterogeneity.

## Discussion

The current study comprehensively examined the causal association of IBD with CeD using summary GWAS data. To the best of our knowledge, this is the first study to use the MR approach to evaluate the bidirectional causal relationship between IBD and CeD. This bidirectional MR study demonstrates that IBD and CD are causally linked to CeD. Surprisingly, our findings implied that UC is a protective factor for CeD, contradicting previous studies. However, we only identified a link between CeD and CD when it came to CeD as an exposure factor, with no indication of a causal relationship with IBD or UC.

The effects of CD and UC on CeD were shown to differ when compared with previous studies. Previous studies have found a higher risk of UC than CD on CeD (Manceñido Marcos et al., 2020; Mårild et al., 2022). Contrary to those observations, the risk effect of CD on CeD was more significant in the current study. In addition, the risk of IBD in patients with CeD are significantly higher than that of CeD in patients with IBD. We compared the results of a recent meta-analysis of observational studies (Pinto-Sanchez et al., 2020) with those of this study. First, we assessed the impact of IBD on the development of CeD. The meta-analysis reported an increased risk of CeD in IBD (IBD RR = 3.96, 95% CI = 2.23–7.02, *p* < 0.00001; CD RR = 3.15, 95% CI = 1.77–5.62, *p* = 0.0001; UC RR = 2.81, 95% CI = 1.82–4.36, *p* < 0.00001). In the current study, it was observed that CeD is affected differently by IBD, CD, and UC (IVW IBD-related SNPs OR = 1.24, 95% CI = 1.16–1.34, *p* = 9.42E-10; CD-related SNPs OR = 1.27, 95% CI = 1.19–1.35, *p* = 3.75E-13; UC-related SNPs OR = 0.90, 95% CI = 0.83–0.98, *p* = 0.017). However, we discovered that UC has a different effect on CeD. The results of the effect of UC on CeD were inconsistent with those of clinical observation studies. We reported that UC might be a protective factor for CeD rather than a risk factor. IBD influences ceD in a way, that is, a mix of CD and UC. Based on the findings of the CD and UC studies, it is hypothesized that CD is the primary causative factor for the causal impact of IBD on CeD in this study. Further, we assessed the impact of CeD on IBD. The meta-analysis reported that patients with CeD have a higher risk of IBD (IBD RR = 9.88, 95% CI = 4.03–24.21, *p* < 0.00001; CD RR = 7.73, 95% CI = 5.09–11.73, *p* < 0.00001; UC RR = 4.08, 95% CI = 2.40–6.95, *p* < 0.00001). According to the findings of the current study, CeD exhibited varying effects on IBD, CD, and UC (IVW IBD OR = 1.00, 95% CI = 0.97–1.04, *p* = 0.900; CD OR = 1.09, 95% CI = 1.05–1.13, *p* = 1.39E-05; UC OR = 0.96, 95% CI = 0.92–1.02, *p* = 0.172). The current study only reported a causal effect of CeD on CD and not the effect of CeD on IBD and UC reported by observational studies. Finally, we reported that the two-way causal effects of IBD and CeD are likewise inconsistent. Following the observational study, the findings revealed that a bidirectional relationship existed between IBD (both CD and UC) and CeD, with the effect of CeD on IBD greater than that of IBD on CeD. In contrast to the previous study, we only discovered a bidirectional causal link between CD and CeD in our investigation, and the influence on CeD was significantly greater than that of CeD on CD in our analysis. The appearance of a causative association contrary to epidemiological research might indicate that the detection rate of CeD in patients with IBD in our study was far from reality or IBD treatment measures are effective in preventing CeD.

The pathophysiology of IBD involves progression from physiological intestinal inflammation to pathological intestinal inflammation. It is believed that the complicated dynamics of luminal microbial communities, host mucosal defense, and response mechanisms have played a significant part in the occurrence of this transition. A study discovered 163 loci, 110 linked to both diseases; the remaining 53 loci were divided into 30 CD-specific and 23 UC-specific categories (Jostins et al., 2012). Risk alleles of two CD genes, *PTPN22* and *NOD2*, exhibited significant (*p* < 0.005) protective effects in UC. Five CD-specific loci and two UC-specific loci were included in the MR analysis. A portion of the varied causal effects of CD and UC on CeD can be attributed to specific genes. Environmental factors such as diet, where high inflammatory potential eating patterns are linked to an elevated risk of CD but not UC (Lo et al., 2020), cannot be overlooked. Dietary habits are linked to CD and CeD but not UC, which may help to explain why CD and UC have different impacts on CeD.

It is thought that CeD is caused by an immune response to gluten, that is, not ideal. Hence, diet is the most critical factor in managing CeD and promoting gluten-free eating is crucial. In most cases, innate and adaptive immunity is implicated at the beginning of immunological responses. Overall, 40 CeD loci have been discovered (Lundin and Wijmenga, 2015). These areas include a high concentration of immune-related genes candidates for inclusion in future studies. Approximately one-third of the loci were observed to have several independent signals, yielding 57 independent CeD link signals. The 39 non-HLA loci in CeD could explain around 14% of the genetic variability in the disease, with the HLA locus accounting for the remaining 40% of the genetic variety.

The etiology of IBD and CeD is yet unknown; however, both diseases have a complicated origin with complex genetic and environmental components. Reaction with gluten and changes in the commensal microbiota appears to be the most relevant ecological variables in CeD and IBD, respectively. Both disorders are characterized by an aberrant immune response and inflammation caused by similar hereditary factors. Overall, 70% of IBD loci (113/163) are shared with other complex illnesses, whereas 12% (20) are shared with CeD. When the number of CeD risk areas is considered a benchmark, 50% of CeD loci are shared with IBD (Pascual et al., 2014). IBD and CeD are both immunological disorders with the same genetic locus, but the triggers for the two diseases are different, with pasta and bacteria being the triggers for IBD and CeD, respectively. The association reported in observational research might be explained by the presence of the same genetic patterns. Even after controlling similar genes and confounding variables, the present study discovered a bidirectional causal connection between CD and CeD. The pathogenic immune response and enhanced intestinal permeability are the same in CD and CeD.

One advantage of the bidirectional approach is that the causation between IBD and CeD may be inferred in both ways. The MR is based on publicly available GWAS summary statistics. It is viable for mining trustworthy genetic data without additional experimental costs. Another benefit is the stratification research we conducted. IBD is divided into two subtypes, CD and UC. Although previous studies have indicated that both CD and UC exhibit similar positive results in IBD, there are variations in the effectiveness of their effects. We discovered that CD and UC had fundamentally distinct relationships with CeD.

This study has some limitations. The samples of IBD and CeD were relatively small, which might have led to misleading positive and negative results. The genetic loci revealed in gut microbiota GWAS are currently limited due to sample size limitations, limiting the statistical power of MR analysis (Swerdlow et al., 2016). To boost the power of testing association, a larger GWAS with more sample size and SNPs is required to replicate MR findings. Although we selected data on cohorts of exclusively European origin, we observed significant variability. We could not eliminate the heterogeneity, making the conclusions less believable. The study had excessive heterogeneity. The study exclusively included participants of European ancestry, and IVs observed in the European population cannot be linked with the non-European population (Lazarus et al., 2002). However, recent epidemiological investigations reveal that non-European groups are gradually becoming the new victims of IBD and CeD.

Moreover, genetic susceptibility and environmental triggers differ between eastern and western populations. Therefore, future research on the eastern population should aim to reproduce the findings on the population of the west on environmental and genetic risk factors and discover new correlations that may be uniquely relevant to local populations. Moreover, MR studies of other ethnic groups are required, which need to be supported by large scale GWAS data.

In conclusion, a bidirectional causal relationship existed between CD and CeD, with CD exerting a greater impact on CeD than the other way around. It is recommended that people with CD undergo routine screening for CeD. On the other hand, the consequences of UC were quite different. Future research on IBD and CeD should distinguish between CD and UC and concentrate on the mechanisms through which CD influences CeD.

## Data Availability

The original contributions presented in the study are included in the article/Supplementary Material, further inquiries can be directed to the corresponding author.
