# Supplementary material for: Inflammatory bowel disease and celiac disease: A bidirectional Mendelian randomization study
Source: Front Genet. 2022 Aug 19;13:928944. doi: 10.3389/fgene.2022.928944 (PMC9437575; doi:10.3389/fgene.2022.928944)
Supplement: Supplementary file 1 [file DataSheet1.docx]

**Supplementary material**

**Supplementary Figure 1. Scatter plots**


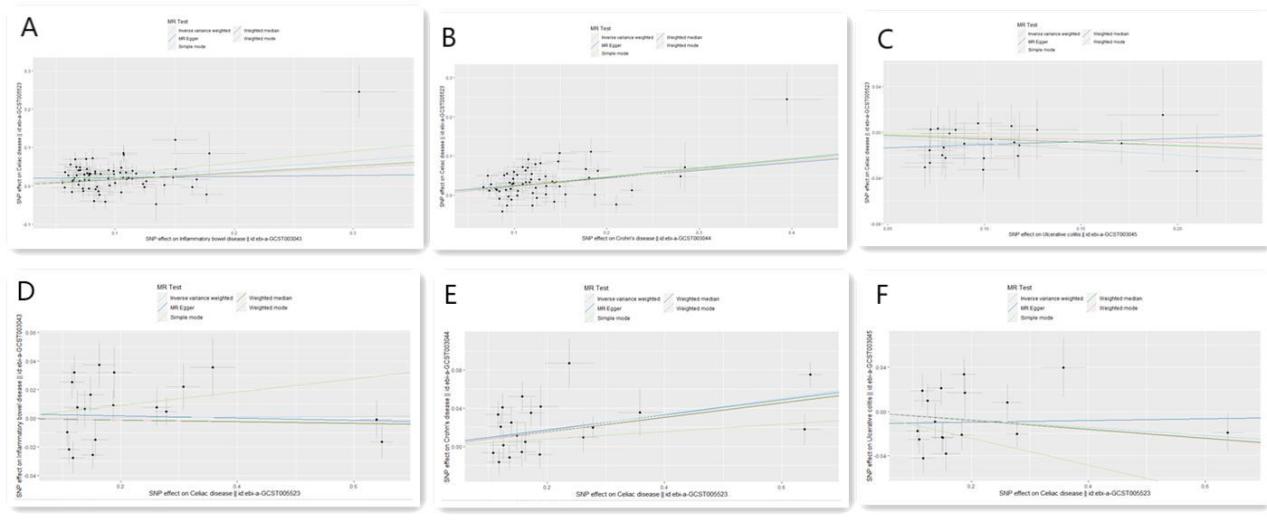


The estimate of intercept can be interpreted as an estimate of the average pleiotropy of all single-nucleotide polymorphisms (SNPs), and the slope coefficient provides an estimate of the bias of the causal effect. (A) IBD on CeD. (B) CD on CeD. (C) UC on CeD. (D) CeD on IBD. (E) CeD on CD. (F) CeD on UC.

**Supplementary Figure 2. Funnel plots**


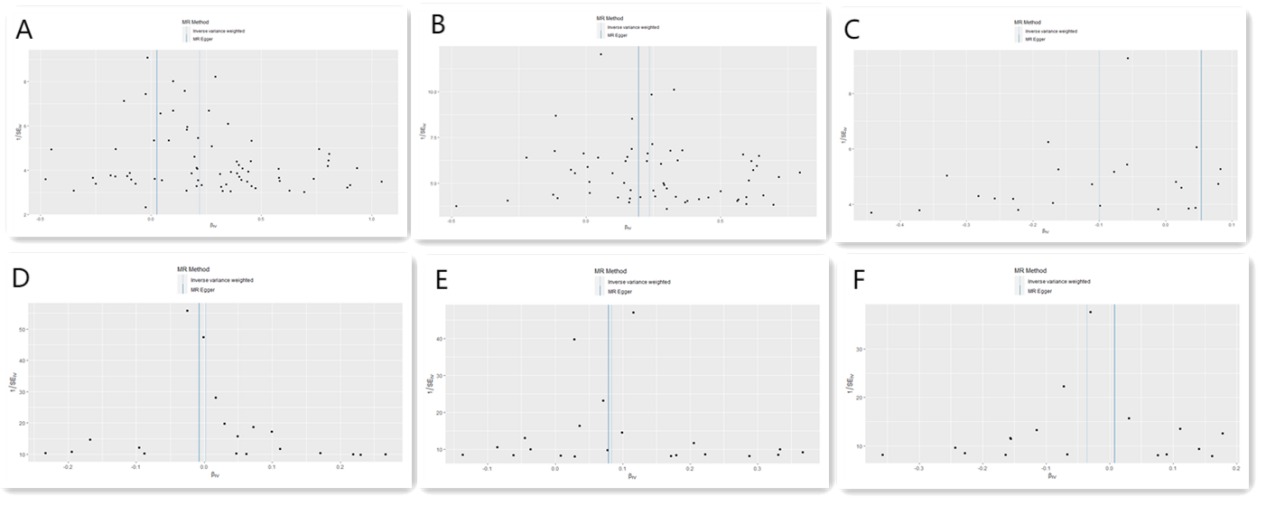


The x-axis represents β, and the y-axis represents 1/SE (standard error). (A) IBD on CeD. (B) CD on CeD. (C) UC on CeD. (D) CeD on IBD. (E) CeD on CD. (F) CeD on UC.

**Supplementary Figure 3. Leave-one-out sensitivity analysis**


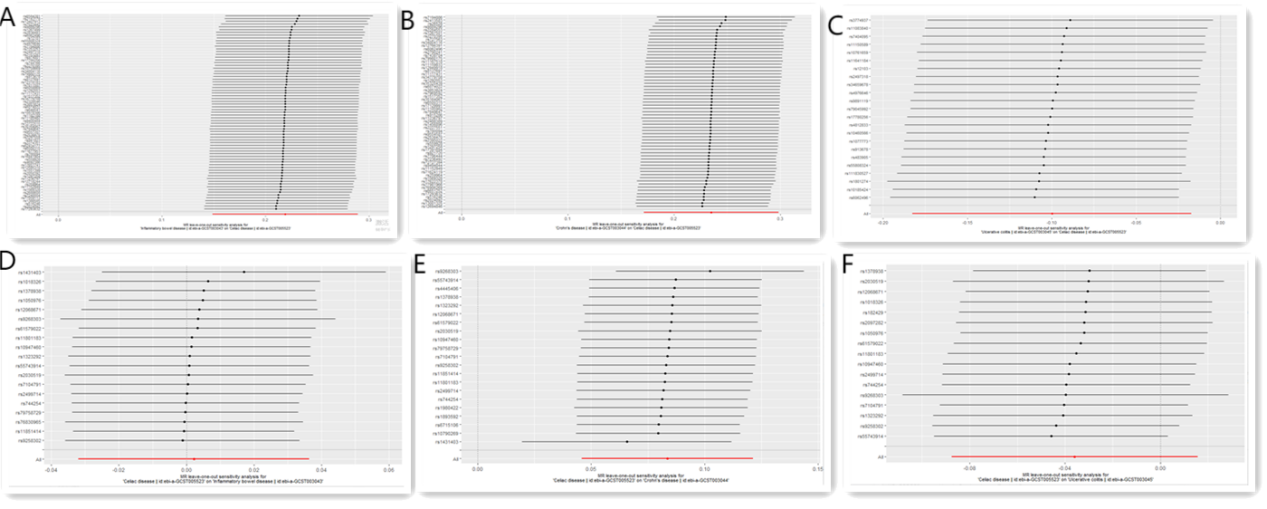


(A) IBD on CeD. (B) CD on CeD. (C) UC on CeD. (D) CeD on IBD. (E) CeD on CD. (F) CeD on UC.

**Supplementary Figure 3. Forest plots**


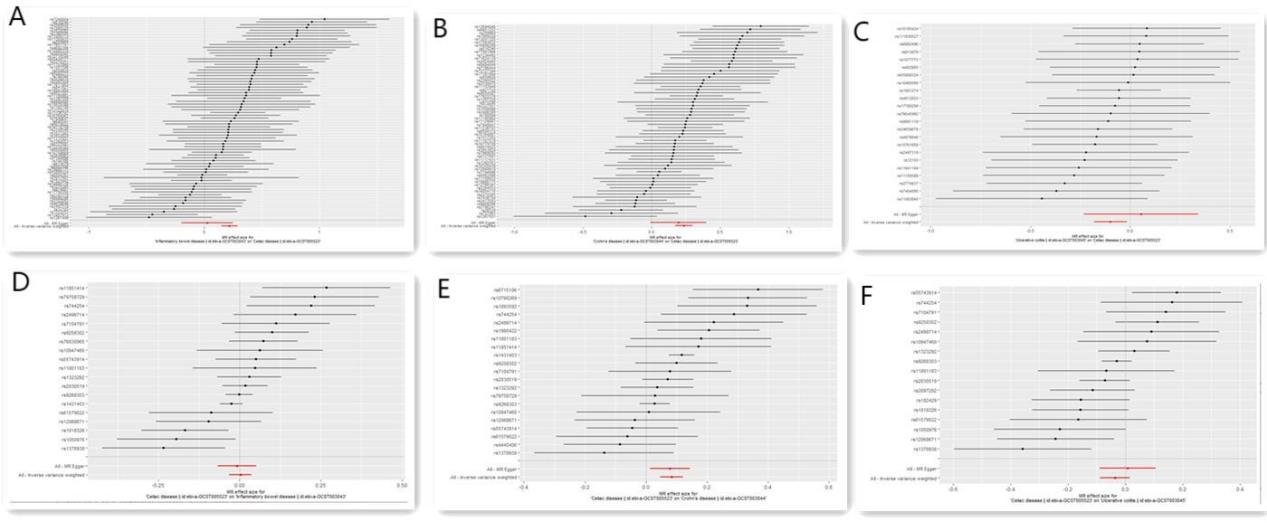


(A) IBD on CeD. (B) CD on CeD. (C) UC on CeD. (D) CeD on IBD. (E) CeD on CD. (F) CeD on UC.
